# Renal Aspergillosis Complicating Renal Allograft Transplantation: A Case Report

**DOI:** 10.7759/cureus.61817

**Published:** 2024-06-06

**Authors:** Sunny Malde, Sushrut Gupta, Pranjal Kashiv, Kapil N Sejpal, Amit S Pasari, Manish Balwani, Vrushali Mahajan, Shubham Dubey, Twinkle Pawar, Vijay Jeyachandran

**Affiliations:** 1 Nephrology, Jawaharlal Nehru Medical College, Datta Meghe Institute of Higher Education and Research, Wardha, IND; 2 Nephrology, Saraswati Kidney Care Center, Nagpur, IND; 3 Nephrology, Saraswati Kidney care Center, Nagpur, IND; 4 Pathology, Alexis Multispeciality Hospital, Nagpur, IND; 5 Medicine, Jawaharlal Nehru Medical College, Datta Meghe Institute of Higher Education and Research, Wardha, IND

**Keywords:** graft nephrectomy, immunosuppression, antibody-mediated rejection, aspergillosis, renal allograft, renal transplantation

## Abstract

Renal aspergillosis is a rare yet potentially devastating complication following renal allograft transplantation. We present the case of a 45-year-old male with a history of crescentic IgA nephropathy who underwent renal allograft transplantation from his mother. Despite initial favorable progress, he developed post-transplant renal dysfunction attributed to active antibody-mediated rejection. Subsequently, he presented with signs of systemic infection and graft dysfunction, leading to the diagnosis of renal aspergillosis. Despite aggressive management, including antifungal therapy and cessation of immunosuppression, the patient progressed to renal graft cortical necrosis, necessitating nephrectomy. This case underscores the challenges in diagnosing and managing renal aspergillosis in transplant recipients and highlights the importance of early recognition and prompt intervention to improve outcomes in such cases.

## Introduction

Renal transplantation is the gold standard treatment for end-stage renal disease (ESRD), offering improved quality of life and increased survival compared to dialysis [1]. However, despite advances in surgical techniques and immunosuppressive regimens, complications following renal allograft transplantation remain a significant concern. One such complication is the development of opportunistic infections, which can lead to graft dysfunction, rejection, and even graft loss.

Immunosuppressive therapy, necessary to prevent graft rejection, predisposes renal transplant recipients to infections by impairing the immune response against pathogens [2]. Among the infectious agents encountered post-transplantation, fungal infections pose a challenge due to their insidious onset, nonspecific clinical manifestations, and resistance to conventional antimicrobial therapy [3]. *Aspergillus* species are ubiquitous molds commonly found in the environment. In immunocompromised individuals, including solid organ transplant recipients, *Aspergillus* can cause invasive infections with high morbidity and mortality rates [4]. Renal aspergillosis following renal transplantation is rare but carries a grave prognosis, often resulting in graft loss and systemic dissemination of the infection [5].

Early diagnosis and prompt initiation of appropriate antifungal therapy are crucial for improving outcomes in patients with renal aspergillosis. However, due to the rarity of this condition and the challenges associated with its diagnosis, renal transplant clinicians must maintain a high index of suspicion and be vigilant in monitoring patients for signs and symptoms suggestive of fungal infection. This case report underscores the importance of recognizing renal aspergillosis as a potential complication following renal allograft transplantation and highlights the complexities involved in its diagnosis and management.

## Case presentation

A 45-year-old male with a history of chronic kidney disease, necessitating maintenance hemodialysis due to crescentic IgA nephropathy, underwent a live-related renal allograft transplantation in October 2023, with his mother as the donor. The donor had no comorbidities and shared blood type B Rh+. Immunological evaluations revealed a 2/6 human leukocyte antigen mismatch, negative HLA mixed antibody screen, and negative T and B cell flow cytometry cross-match. Intraoperatively, the patient received induction therapy with Inj. antithymocyte globulin (1.5mg/kg) and a methylprednisolone pulse of 500mg. The cold ischemia time was 45 minutes. Upon unclamping, he exhibited a urine output of 460mL within the first hour. Immunosuppressive medication included tacrolimus (TAC) at 4mg/day and mycophenolate mofetil at 2gm/day. Pretransplant creatinine levels were at 6mg/dL. Initially, he demonstrated favorable urine output and decreasing creatinine levels until day 8, when his creatinine level rose to 1.6mg/dL, followed by a subsequent increase to 2mg/dL, prompting investigations for post-transplant renal dysfunction. Doppler ultrasound confirmed a well-perfused graft without hydronephrosis; TAC levels remained normal.

On the ninth post-operative day, a graft biopsy revealed active antibody-mediated rejection with mild glomerulitis and C4d positivity. Treatment included four sessions of plasmapheresis and Inj. rituximab (200mg) administered on November 8, 2023. His creatinine levels were at 1.3mg/dL on discharge, and he continued to progress well during subsequent outpatient visits. However, on December 8, 2023, he was readmitted due to swelling in both lower limbs, reduced urine output, and loose stools, with a creatinine level of 2.9mg/dL. Despite normal TAC levels and Doppler ultrasound findings, a subsequent graft biopsy on December 12, 2023, revealed microvascular inflammation with C4d positivity, active antibody-mediated rejection, mild interstitial fibrosis and tubular atrophy (IFTA), and microabscess formation with fungal elements in the allograft.

**Figure 1:**
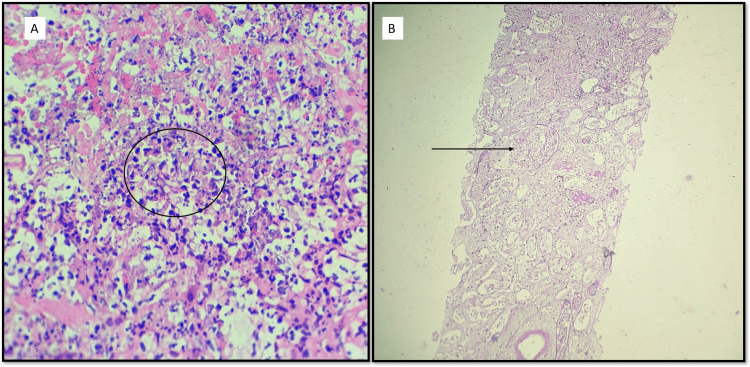
(A) H&E (40X). Microphotograph showing microabscess in renal cortical tissue with prominent karyorrhectic debris. The circle highlights the fungal hyphae, which are long and septate. (B) PAS (40X). Microphotograph showing renal cortical tissue with diffuse necrosis. The arrow points towards a necrosed glomerulus. Only ghost outlines of the glomeruli, and surrounding tubules and vessels could be appreciated.

Further interventions, including eight cycles of plasmapheresis, failed to improve renal function or urine output. Subsequently, on December 24, 2023, the patient developed sudden-onset breathlessness, leading to hemodialysis and a diagnosis of acute respiratory distress syndrome. Immunomodulatory medications were ceased due to an active infection, and treatment for graft pyelonephritis commenced with broad-spectrum antibiotics and voriconazole. Despite these efforts, the patient's condition worsened, ultimately culminating in renal graft cortical necrosis. Following multiple hemodialysis sessions, stabilization was achieved for graft nephrectomy. Histopathological examination of the explanted graft revealed coagulative necrosis consistent with renal aspergillosis. Oral voriconazole therapy was initiated, and the patient was discharged in stable condition on the eighth day post-nephrectomy (Figure 1). The patient continues biweekly hemodialysis without complications, maintaining a residual urine output of 50mL/day.

## Discussion

Renal transplantation is a life-saving intervention for individuals with ESRD, offering improved quality of life and increased longevity. However, it has its challenges, including the risk of infectious complications. Fungal infections in renal transplant recipients are relatively rare but can lead to significant morbidity and mortality if not promptly diagnosed and treated. In this case report, we discuss the development of renal aspergillosis in a patient post-renal allograft transplantation. Fungal infections following renal transplantation typically occur due to immunosuppression, prolonged hospitalization, and prior fungal colonization [3]. *Aspergillus* species are among the most common fungal pathogens implicated in these infections, with *Aspergillus fumigatus* being the predominant species [6]. The exact mechanism of acquisition of *Aspergillus* in transplant recipients remains unclear, but inhalation of airborne spores is believed to be the primary route of entry [7].

One of the challenges in diagnosing fungal infections in renal transplant recipients is the nonspecific clinical presentation. Patients may present with fever, graft dysfunction, and systemic symptoms, which can overlap with other post-transplant complications [8]. As seen in our case, the initial presentation of reduced urine output and rising creatinine levels could have been attributed to rejection episodes, highlighting the importance of maintaining a high index of suspicion for fungal infections in these patients.

Early diagnosis of fungal infections is essential for promptly initiating appropriate antifungal therapy. In our case, the diagnosis of renal aspergillosis was confirmed through graft biopsy, which revealed characteristic fungal elements. However, the biopsy was performed after the patient had already developed significant graft dysfunction, underscoring the need for better diagnostic modalities to enable earlier detection of fungal infections in transplant recipients. Treatment of renal aspergillosis typically involves a combination of antifungal therapy and, in severe cases, surgical intervention. Voriconazole is the preferred antifungal agent for treating invasive aspergillosis, as it is superior to amphotericin B in terms of efficacy and safety [9]. In our case, oral voriconazole therapy was initiated following the diagnosis of renal aspergillosis, but unfortunately, the patient's condition continued to deteriorate, ultimately necessitating graft nephrectomy.

Graft nephrectomy is considered in cases of irreversible graft dysfunction or when there is a risk of disseminated infection [10]. In our patient, despite aggressive antifungal therapy and cessation of immunomodulatory medications, the progression to renal graft cortical necrosis necessitated nephrectomy. This decision was made to prevent further infection dissemination and stabilize the patient's clinical condition.

## Conclusions

In conclusion, the presented case highlights the grave implications of renal aspergillosis as a rare but formidable complication following renal allograft transplantation. Despite advancements in immunosuppressive strategies and antifungal therapies, the morbidity and mortality associated with this infection remain significant. The case underscores the critical need for vigilance in suspecting fungal infections in transplant recipients presenting with graft dysfunction, especially when accompanied by systemic signs of infection. Furthermore, it emphasizes the challenges in managing such cases, as illustrated by the progression to renal graft cortical necrosis despite aggressive therapeutic interventions. Moving forward, ongoing research efforts should focus on enhancing preventive strategies, improving early detection methods, and optimizing treatment approaches to address the complexities of fungal infections in transplant recipients and ultimately improve patient outcomes.
